# (Anthracen-9-yl)(piperidin-1-yl)­methanone

**DOI:** 10.1107/S1600536808033205

**Published:** 2008-10-15

**Authors:** Hua-You Hu, Yu-Cheng Huang, Hai-Tao Yu, Yan Zhang

**Affiliations:** a Department of Chemistry and Chemical Engineering, Southeast University, Nanjing 211189, People’s Republic of China; b School of Chemistry and Chemical Engineering, Key Laboratory of Analytical Chemistry for Life Science, Ministry of Education, Nanjing University, Nanjing 210093, People’s Republic of China

## Abstract

The title compound, C_20_H_19_NO, is a substructure of CP-640186, a potent inhibitor of mammalian acetyl-coenzyme A carboxyl­ases. In the crystal structure, the amide group forms a dihedral angle of 87.0 (1)° with the plane of the anthracene unit and the piperidine ring adopts a chair conformation. Mol­ecules are arranged into layers parallel to (100) and adjacent anthracene units within layers form dihedral angles of 13.2 (1)°. C—H⋯O inter­actions from the piperidine rings to the C=O group of the amide are observed between layers.

## Related literature

For further information regarding CP-640186, see: Harwood *et al.* (2003 ▶); Zhang *et al.* (2004 ▶).
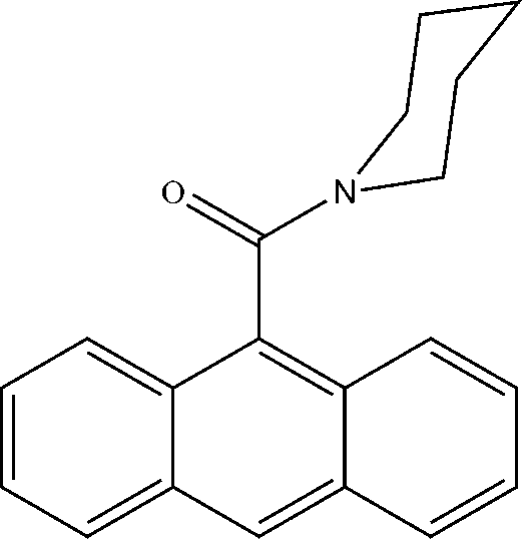


## Experimental

### 

#### Crystal data

C_20_H_19_NO
*M* *_r_* = 289.36
Monoclinic, 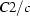

*a* = 26.393 (5) Å
*b* = 7.3950 (15) Å
*c* = 18.213 (4) Å
β = 120.29 (3)°
*V* = 3069.5 (14) Å^3^
*Z* = 8
Mo *K*α radiation
μ = 0.08 mm^−1^
*T* = 293 (2) K
0.30 × 0.10 × 0.10 mm

#### Data collection

Bruker SMART APEX CCD diffractometer
Absorption correction: multi-scan (*SADABS*; Bruker, 2000 ▶) *T*_min_ = 0.977, *T*_max_ = 0.992
2828 measured reflections
2762 independent reflections
1678 reflections with *I* > 2σ(*I*)
*R*_int_ = 0.025

#### Refinement

*R*[*F* ^2^ > 2σ(*F* ^2^)] = 0.081
*wR*(*F* ^2^) = 0.278
*S* = 1.09
2762 reflections
199 parameters
H-atom parameters constrained
Δρ_max_ = 0.32 e Å^−3^
Δρ_min_ = −0.29 e Å^−3^

### 

Data collection: *SMART* (Bruker, 2000 ▶); cell refinement: *SAINT* (Bruker, 2000 ▶); data reduction: *SAINT*; program(s) used to solve structure: *SHELXS97* (Sheldrick, 2008 ▶); program(s) used to refine structure: *SHELXL97* (Sheldrick, 2008 ▶); molecular graphics: *SHELXTL* (Sheldrick, 2008 ▶); software used to prepare material for publication: *SHELXTL*.

## Supplementary Material

Crystal structure: contains datablocks I, global. DOI: 10.1107/S1600536808033205/bi2300sup1.cif

Structure factors: contains datablocks I. DOI: 10.1107/S1600536808033205/bi2300Isup2.hkl

Additional supplementary materials:  crystallographic information; 3D view; checkCIF report

## Figures and Tables

**Table 1:** Hydrogen-bond geometry (Å, °)

*D*—H⋯*A*	*D*—H	H⋯*A*	*D*⋯*A*	*D*—H⋯*A*
C17—H17*B*⋯O1^i^	0.97	2.41	3.342 (5)	162
C20—H20*A*⋯O1^ii^	0.97	2.71	3.557 (5)	146

Symmetry codes: (i) 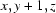; (ii) 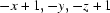.

**Fig. 1.**
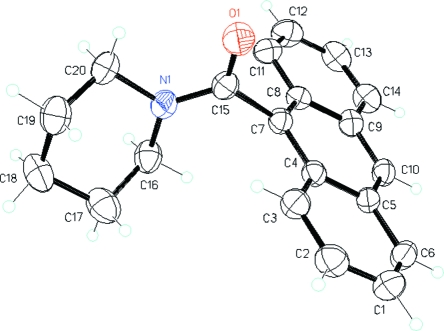
The molecular structure of the title compound with displacement ellipsoids shown at 30% probability for non-H atoms.

## supplementary crystallographic information

### Comment

Inhibition of acetyl-CoA carboxylase (ACC), with its resultant inhibition of fatty acid synthesis and stimulation of fatty acid oxidation, has the potential to affect favourably the multitude of cardiovascular risk factors associated with the metabolic syndrome. Recent findings reported by Pfizer researchers show that the isozyme-nonselective ACC inhibitor CP-640186 inhibits both the lipogenic tissue isozyme (ACC1) and the oxidative tissue isozyme (ACC2) (Harwood *et al.*, 2003). The title compound is a sub-structure of CP-640186 (see Zhang *et al.*, 2004).

### Experimental

A mixture of 9-carbonyl anthracene (1 mmol) and piperidine (1.2 mmol) with 1.5 mmol DCC (DCC = *N*,*N*'-dicyclohexyl-carbodiimide) was stirred in 5 ml CH_2_Cl_2_ at room temperature for 1 h to yield the title compound. Crystals were obtained from acetone/petroleum ether.

### Refinement

H atoms were positioned geometrically and refined using a riding model with C—H = 0.93–0.97 Å and with *U*_iso_(H) = 1.2*U*_eq_(C).

### Crystal data

**Table:**

C_20_H_19_NO	*F*(000) = 1232
*M**_r_* = 289.36	*D*_x_ = 1.252 Mg m^−^^3^
Monoclinic, *C*2/*c*	Mo *K*α radiation, λ = 0.71073 Å
Hall symbol: -C 2yc	Cell parameters from 781 reflections
*a* = 26.393 (5) Å	θ = 2.4–28.0°
*b* = 7.3950 (15) Å	µ = 0.08 mm^−^^1^
*c* = 18.213 (4) Å	*T* = 293 K
β = 120.29 (3)°	Block, yellow
*V* = 3069.5 (14) Å^3^	0.30 × 0.10 × 0.10 mm
*Z* = 8	

### Data collection

**Table:**

Bruker SMART APEX CCD diffractometer	2762 independent reflections
Radiation source: fine-focus sealed tube	1678 reflections with *I* > 2σ(*I*)
graphite	*R*_int_ = 0.025
φ and ω scans	θ_max_ = 25.2°, θ_min_ = 1.8°
Absorption correction: multi-scan (SADABS; Bruker, 2000)	*h* = −31→27
*T*_min_ = 0.977, *T*_max_ = 0.992	*k* = 0→8
2828 measured reflections	*l* = 0→21

### Refinement

**Table:**

Refinement on *F*^2^	Primary atom site location: structure-invariant direct methods
Least-squares matrix: full	Secondary atom site location: difference Fourier map
*R*[*F*^2^ > 2σ(*F*^2^)] = 0.081	Hydrogen site location: inferred from neighbouring sites
*wR*(*F*^2^) = 0.278	H-atom parameters constrained
*S* = 1.09	*w* = 1/[σ^2^(*F*_o_^2^) + (0.1523*P*)^2^ + 2.2222*P*] where *P* = (*F*_o_^2^ + 2*F*_c_^2^)/3
2762 reflections	(Δ/σ)_max_ < 0.001
199 parameters	Δρ_max_ = 0.32 e Å^−^^3^
0 restraints	Δρ_min_ = −0.28 e Å^−^^3^

### Special details

**Table:**

Geometry. All e.s.d.'s (except the e.s.d. in the dihedral angle between two l.s. planes) are estimated using the full covariance matrix. The cell e.s.d.'s are taken into account individually in the estimation of e.s.d.'s in distances, angles and torsion angles; correlations between e.s.d.'s in cell parameters are only used when they are defined by crystal symmetry. An approximate (isotropic) treatment of cell e.s.d.'s is used for estimating e.s.d.'s involving l.s. planes.
Refinement. Refinement of *F*^2^ against ALL reflections. The weighted *R*-factor *wR* and goodness of fit *S* are based on *F*^2^, conventional *R*-factors *R* are based on *F*, with *F* set to zero for negative *F*^2^. The threshold expression of *F*^2^ > σ(*F*^2^) is used only for calculating *R*-factors(gt) *etc*. and is not relevant to the choice of reflections for refinement. *R*-factors based on *F*^2^ are statistically about twice as large as those based on *F*, and *R*- factors based on ALL data will be even larger.

### Fractional atomic coordinates and isotropic or equivalent isotropic displacement parameters (Å^2^)

**Table:**

	*x*	*y*	*z*	*U*_iso_*/*U*_eq_	
C1	0.83354 (17)	0.1537 (5)	0.6866 (3)	0.0733 (11)	
H1A	0.8709	0.1589	0.6936	0.088*	
C2	0.78453 (17)	0.1309 (5)	0.6048 (3)	0.0680 (10)	
H2A	0.7896	0.1254	0.5579	0.082*	
C3	0.72989 (15)	0.1169 (5)	0.5938 (2)	0.0577 (9)	
H3A	0.6980	0.0993	0.5394	0.069*	
C4	0.72079 (13)	0.1286 (4)	0.66384 (19)	0.0472 (8)	
C5	0.77079 (14)	0.1544 (4)	0.7464 (2)	0.0504 (8)	
C6	0.82684 (15)	0.1679 (5)	0.7541 (2)	0.0611 (9)	
H6A	0.8596	0.1871	0.8074	0.073*	
C7	0.66500 (13)	0.1159 (4)	0.65502 (19)	0.0480 (8)	
C8	0.65705 (14)	0.1315 (4)	0.7257 (2)	0.0478 (8)	
C9	0.70746 (15)	0.1503 (4)	0.8081 (2)	0.0534 (8)	
C10	0.76293 (14)	0.1644 (4)	0.8162 (2)	0.0538 (9)	
H10A	0.7956	0.1809	0.8699	0.065*	
C11	0.60162 (16)	0.1229 (5)	0.7191 (3)	0.0626 (10)	
H11A	0.5682	0.1098	0.6658	0.075*	
C12	0.59630 (19)	0.1335 (5)	0.7889 (3)	0.0751 (12)	
H12A	0.5591	0.1308	0.7826	0.090*	
C13	0.6455 (2)	0.1485 (5)	0.8706 (3)	0.0718 (11)	
H13A	0.6409	0.1535	0.9180	0.086*	
C14	0.69916 (19)	0.1556 (5)	0.8799 (2)	0.0677 (10)	
H14A	0.7317	0.1642	0.9343	0.081*	
C15	0.61267 (14)	0.0678 (5)	0.5689 (2)	0.0524 (8)	
C16	0.59033 (17)	0.3956 (5)	0.5392 (2)	0.0693 (11)	
H16A	0.6271	0.4108	0.5922	0.083*	
H16B	0.5593	0.4463	0.5465	0.083*	
C17	0.5933 (2)	0.4948 (6)	0.4705 (3)	0.0870 (13)	
H17A	0.6274	0.4549	0.4680	0.104*	
H17B	0.5975	0.6232	0.4831	0.104*	
C18	0.5372 (2)	0.4617 (6)	0.3833 (3)	0.0848 (13)	
H18A	0.5036	0.5164	0.3828	0.102*	
H18B	0.5416	0.5166	0.3384	0.102*	
C19	0.52736 (19)	0.2577 (7)	0.3679 (2)	0.0861 (13)	
H19A	0.4905	0.2372	0.3156	0.103*	
H19B	0.5585	0.2072	0.3607	0.103*	
C20	0.52607 (17)	0.1630 (6)	0.4382 (2)	0.0798 (12)	
H20A	0.4917	0.2008	0.4406	0.096*	
H20B	0.5233	0.0337	0.4281	0.096*	
N1	0.57930 (13)	0.2033 (4)	0.51987 (17)	0.0652 (9)	
O1	0.60244 (11)	−0.0901 (3)	0.54889 (16)	0.0754 (9)	

### Atomic displacement parameters (Å^2^)

**Table:**

	*U*^11^	*U*^22^	*U*^33^	*U*^12^	*U*^13^	*U*^23^
C1	0.058 (2)	0.078 (3)	0.090 (3)	0.0004 (18)	0.042 (2)	0.008 (2)
C2	0.076 (3)	0.071 (2)	0.071 (2)	0.0052 (19)	0.048 (2)	0.0061 (19)
C3	0.059 (2)	0.063 (2)	0.0532 (19)	0.0000 (16)	0.0293 (17)	0.0003 (15)
C4	0.0487 (18)	0.0408 (16)	0.0483 (18)	0.0024 (13)	0.0216 (15)	0.0022 (13)
C5	0.0448 (17)	0.0440 (17)	0.0513 (18)	0.0004 (13)	0.0160 (15)	0.0049 (14)
C6	0.0438 (19)	0.060 (2)	0.068 (2)	0.0006 (15)	0.0189 (17)	−0.0010 (17)
C7	0.0475 (18)	0.0449 (18)	0.0438 (17)	0.0006 (13)	0.0173 (14)	0.0000 (13)
C8	0.0527 (19)	0.0420 (17)	0.0519 (18)	−0.0014 (13)	0.0286 (16)	0.0008 (13)
C9	0.059 (2)	0.0469 (18)	0.0481 (18)	0.0035 (15)	0.0229 (16)	−0.0049 (14)
C10	0.0507 (19)	0.0506 (19)	0.0459 (18)	0.0015 (14)	0.0139 (15)	0.0006 (14)
C11	0.059 (2)	0.062 (2)	0.071 (2)	−0.0058 (16)	0.0350 (19)	−0.0031 (17)
C12	0.078 (3)	0.077 (3)	0.093 (3)	−0.001 (2)	0.060 (3)	−0.002 (2)
C13	0.099 (3)	0.070 (2)	0.068 (2)	0.011 (2)	0.058 (2)	0.0023 (19)
C14	0.084 (3)	0.068 (2)	0.054 (2)	−0.0005 (19)	0.036 (2)	−0.0040 (17)
C15	0.0497 (18)	0.057 (2)	0.0485 (18)	−0.0007 (15)	0.0238 (15)	−0.0044 (16)
C16	0.070 (2)	0.066 (2)	0.057 (2)	0.0051 (18)	0.0207 (19)	−0.0063 (18)
C17	0.112 (4)	0.063 (3)	0.085 (3)	0.003 (2)	0.049 (3)	0.003 (2)
C18	0.110 (3)	0.076 (3)	0.064 (2)	0.013 (2)	0.040 (2)	0.016 (2)
C19	0.086 (3)	0.103 (4)	0.050 (2)	−0.007 (2)	0.020 (2)	−0.003 (2)
C20	0.060 (2)	0.092 (3)	0.058 (2)	−0.012 (2)	0.0075 (19)	0.003 (2)
N1	0.0575 (18)	0.0608 (19)	0.0531 (17)	−0.0090 (14)	0.0100 (14)	0.0008 (14)
O1	0.0812 (19)	0.0499 (15)	0.0646 (16)	−0.0093 (12)	0.0144 (14)	−0.0150 (12)

### Geometric parameters (Å, °)

**Table:**

C1—C6	1.333 (5)	C12—H12A	0.930
C1—C2	1.406 (5)	C13—C14	1.339 (6)
C1—H1A	0.930	C13—H13A	0.930
C2—C3	1.356 (5)	C14—H14A	0.930
C2—H2A	0.930	C15—O1	1.213 (4)
C3—C4	1.415 (4)	C15—N1	1.336 (4)
C3—H3A	0.930	C16—N1	1.458 (5)
C4—C7	1.401 (4)	C16—C17	1.486 (6)
C4—C5	1.427 (4)	C16—H16A	0.970
C5—C10	1.389 (5)	C16—H16B	0.970
C5—C6	1.417 (5)	C17—C18	1.549 (6)
C6—H6A	0.930	C17—H17A	0.970
C7—C8	1.409 (4)	C17—H17B	0.970
C7—C15	1.519 (4)	C18—C19	1.532 (6)
C8—C11	1.407 (5)	C18—H18A	0.970
C8—C9	1.424 (5)	C18—H18B	0.970
C9—C10	1.399 (5)	C19—C20	1.476 (6)
C9—C14	1.433 (5)	C19—H19A	0.970
C10—H10A	0.930	C19—H19B	0.970
C11—C12	1.351 (5)	C20—N1	1.472 (4)
C11—H11A	0.930	C20—H20A	0.970
C12—C13	1.401 (6)	C20—H20B	0.970
			
C6—C1—C2	120.5 (3)	C13—C14—C9	121.5 (4)
C6—C1—H1A	119.7	C13—C14—H14A	119.3
C2—C1—H1A	119.7	C9—C14—H14A	119.3
C3—C2—C1	120.5 (4)	O1—C15—N1	123.3 (3)
C3—C2—H2A	119.8	O1—C15—C7	119.0 (3)
C1—C2—H2A	119.8	N1—C15—C7	117.7 (3)
C2—C3—C4	120.9 (3)	N1—C16—C17	111.5 (3)
C2—C3—H3A	119.5	N1—C16—H16A	109.3
C4—C3—H3A	119.5	C17—C16—H16A	109.3
C7—C4—C3	122.6 (3)	N1—C16—H16B	109.3
C7—C4—C5	119.3 (3)	C17—C16—H16B	109.3
C3—C4—C5	118.1 (3)	H16A—C16—H16B	108.0
C10—C5—C6	122.4 (3)	C16—C17—C18	110.9 (4)
C10—C5—C4	119.0 (3)	C16—C17—H17A	109.5
C6—C5—C4	118.6 (3)	C18—C17—H17A	109.5
C1—C6—C5	121.4 (3)	C16—C17—H17B	109.5
C1—C6—H6A	119.3	C18—C17—H17B	109.5
C5—C6—H6A	119.3	H17A—C17—H17B	108.0
C4—C7—C8	121.4 (3)	C19—C18—C17	109.2 (3)
C4—C7—C15	119.3 (3)	C19—C18—H18A	109.8
C8—C7—C15	119.0 (3)	C17—C18—H18A	109.8
C11—C8—C7	123.0 (3)	C19—C18—H18B	109.8
C11—C8—C9	118.2 (3)	C17—C18—H18B	109.8
C7—C8—C9	118.7 (3)	H18A—C18—H18B	108.3
C10—C9—C8	119.4 (3)	C20—C19—C18	112.6 (4)
C10—C9—C14	122.4 (3)	C20—C19—H19A	109.1
C8—C9—C14	118.2 (3)	C18—C19—H19A	109.1
C5—C10—C9	122.0 (3)	C20—C19—H19B	109.1
C5—C10—H10A	119.0	C18—C19—H19B	109.1
C9—C10—H10A	119.0	H19A—C19—H19B	107.8
C12—C11—C8	120.9 (4)	N1—C20—C19	110.7 (3)
C12—C11—H11A	119.5	N1—C20—H20A	109.5
C8—C11—H11A	119.5	C19—C20—H20A	109.5
C11—C12—C13	121.6 (4)	N1—C20—H20B	109.5
C11—C12—H12A	119.2	C19—C20—H20B	109.5
C13—C12—H12A	119.2	H20A—C20—H20B	108.1
C14—C13—C12	119.5 (4)	C15—N1—C16	125.9 (3)
C14—C13—H13A	120.3	C15—N1—C20	119.7 (3)
C12—C13—H13A	120.3	C16—N1—C20	114.4 (3)
			
C6—C1—C2—C3	−2.2 (6)	C8—C9—C10—C5	−2.3 (5)
C1—C2—C3—C4	1.3 (5)	C14—C9—C10—C5	178.3 (3)
C2—C3—C4—C7	179.6 (3)	C7—C8—C11—C12	178.1 (3)
C2—C3—C4—C5	−0.4 (5)	C9—C8—C11—C12	0.5 (5)
C7—C4—C5—C10	0.7 (4)	C8—C11—C12—C13	−1.7 (6)
C3—C4—C5—C10	−179.3 (3)	C11—C12—C13—C14	1.1 (6)
C7—C4—C5—C6	−179.7 (3)	C12—C13—C14—C9	0.7 (6)
C3—C4—C5—C6	0.3 (4)	C10—C9—C14—C13	177.5 (3)
C2—C1—C6—C5	2.2 (6)	C8—C9—C14—C13	−1.9 (5)
C10—C5—C6—C1	178.4 (3)	C4—C7—C15—O1	84.2 (4)
C4—C5—C6—C1	−1.2 (5)	C8—C7—C15—O1	−90.4 (4)
C3—C4—C7—C8	−178.8 (3)	C4—C7—C15—N1	−97.4 (4)
C5—C4—C7—C8	1.2 (4)	C8—C7—C15—N1	88.0 (4)
C3—C4—C7—C15	6.7 (5)	N1—C16—C17—C18	54.6 (5)
C5—C4—C7—C15	−173.3 (3)	C16—C17—C18—C19	−53.5 (5)
C4—C7—C8—C11	178.8 (3)	C17—C18—C19—C20	53.9 (5)
C15—C7—C8—C11	−6.7 (5)	C18—C19—C20—N1	−54.1 (5)
C4—C7—C8—C9	−3.6 (4)	O1—C15—N1—C16	−177.9 (4)
C15—C7—C8—C9	170.9 (3)	C7—C15—N1—C16	3.7 (5)
C11—C8—C9—C10	−178.2 (3)	O1—C15—N1—C20	2.1 (5)
C7—C8—C9—C10	4.1 (4)	C7—C15—N1—C20	−176.3 (3)
C11—C8—C9—C14	1.3 (4)	C17—C16—N1—C15	124.0 (4)
C7—C8—C9—C14	−176.5 (3)	C17—C16—N1—C20	−56.0 (5)
C6—C5—C10—C9	−179.8 (3)	C19—C20—N1—C15	−125.0 (4)
C4—C5—C10—C9	−0.1 (5)	C19—C20—N1—C16	55.1 (5)

### Hydrogen-bond geometry (Å, °)

**Table:**

*D*—H···*A*	*D*—H	H···*A*	*D*···*A*	*D*—H···*A*
C17—H17B···O1^i^	0.97	2.41	3.342 (5)	162
C20—H20A···O1^ii^	0.97	2.71	3.557 (5)	146

Symmetry codes: (i) *x*, *y*+1, *z*; (ii) −*x*+1, −*y*, −*z*+1.
